# Delayed Fracture Healing and Increased Callus Adiposity in a C57BL/6J Murine Model of Obesity-Associated Type 2 Diabetes Mellitus

**DOI:** 10.1371/journal.pone.0099656

**Published:** 2014-06-09

**Authors:** Matthew L. Brown, Kiminori Yukata, Christopher W. Farnsworth, Ding-Geng Chen, Hani Awad, Matthew J. Hilton, Regis J. O'Keefe, Lianping Xing, Robert A. Mooney, Michael J. Zuscik

**Affiliations:** 1 Center for Musculoskeletal Research, University of Rochester Medical Center, Rochester, New York, United States of America; 2 School of Medicine and Dentistry, University of Rochester Medical Center, Rochester, New York, United States of America; 3 Department of Biostatistics and Computational Biology, University of Rochester Medical Center, Rochester, New York, United States of America; 4 Department of Orthopaedics and Rehabilitation, University of Rochester Medical Center, Rochester, New York, United States of America; 5 Department of Biomechanical Engineering, University of Rochester, Rochester, New York, United States of America; 6 Department of Pathology and Laboratory Medicine, University of Rochester Medical Center, Rochester, New York, United States of America; Oklahoma State University, United States of America

## Abstract

**Introduction:**

Impaired healing and non-union of skeletal fractures is a major public health problem, with morbidity exacerbated in patients with diabetes mellitus (DM). DM is prevalent worldwide and affects approximately 25.8 million US adults, with >90% having obesity-related type 2 DM (T2DM). While fracture healing in type 1 DM (T1DM) has been studied using animal models, an investigation into delayed healing in an animal model of T2DM has not yet been performed.

**Methods:**

Male C57BL/6J mice at 5 weeks of age were placed on either a control lean diet or an experimental high-fat diet (HFD) for 12 weeks. A mid-diaphyseal open tibia fracture was induced at 17 weeks of age and a spinal needle was used for intra-medullary fixation. Mice were sacrificed at days 7, 10, 14, 21, 28, and 35 for micro-computed tomography (μCT), histology-based histomorphometry and molecular analyses, and biomechanical testing.

**Results:**

HFD-fed mice displayed increased body weight and impaired glucose tolerance, both characteristic of T2DM. Compared to control mice, HFD-fed mice with tibia fractures showed significantly (*p*<0.001) decreased woven bone at day 28 by histomorphometry and significantly (*p*<0.01) decreased callus bone volume at day 21 by μCT. Interestingly, fracture calluses contained markedly increased adiposity in HFD-fed mice at days 21, 28, and 35. HFD-fed mice also showed increased PPARγ immunohistochemical staining at day 14. Finally, calluses from HFD-fed mice at day 35 showed significantly (*p*<0.01) reduced torsional rigidity compared to controls.

**Discussion:**

Our murine model of T2DM demonstrated delayed fracture healing and weakened biomechanical properties, and was distinctly characterized by increased callus adiposity. This suggests altered mesenchymal stem cell fate determination with a shift to the adipocyte lineage at the expense of the osteoblast lineage. The up-regulation of PPARγ in fracture calluses of HFD-fed mice is likely involved in the proposed fate switching.

## Introduction

Delayed and failed fracture repair and bone healing are major public health issues in the United States. Musculoskeletal disease impairs the performance of normal daily activities, compromises the performance of other organ systems, and impairs psychological health and social function [1]–[7]. While all patients with bone injury are affected, morbidity is significantly exacerbated in patients with diabetes, which is recognized clinically to delay bone healing and contribute to increased incidence of fibrous non-union (i.e. failure to heal) [8], [9].

Diabetes mellitus (DM) is one of the most common diseases in the United States. The Centers for Disease Control and Prevention (CDC) estimated that as of 2011, approximately 11.3% of the United States population ≥20 years of age were symptomatic diabetics, which includes a total of approximately 25.8 million people – 18.8 million diagnosed and 7.0 million undiagnosed [10]. With DM incidence doubling between 1990 and 2005, the CDC has declared it an epidemic in our society. Despite this warning, the incidence of DM in the US in 2010 was approximately 1.9 million cases [10].

Two types of diabetes exist, each with a distinct etiology. In type 1 DM (T1DM) the loss of insulin-secreting pancreatic β cells causes an absolute insulin insufficiency, resulting from an autoimmune process that has a strong genetic component. The hyperglycemia that occurs in T1DM causes long-term damage to tissues and organs. Type 2 DM (T2DM) is more prevalent in the United States and accounts for >90% of all patients with diabetes [10]. Patients with T2DM, which was previously referred to as non-insulin dependent diabetes (NIDDM) or adult onset diabetes, have elevated blood glucose levels secondary to insulin insensitivity and relative insulin deficiency. Importantly, while T2DM also has a genetic component, it has been more widely associated with obesity and consumption of a high fat diet that is rich in unsaturated fats. Thus, lifestyle and dietary modifications provide an opportunity to mollify the incidence, prevalence and societal burden of T2DM overall.

Rodent models of fracture healing in diabetes have focused on T1DM, which was either caused by an autoimmune destruction of pancreatic β cells or was induced via administration of streptozotocin (STZ) to these cells. Fracture repair studies in these models of T1DM have led to the discovery of several tissue-level defects that alter the normal bone healing program. Normal bone healing involves recruitment and proliferation of stem cell populations at the injury site, chondrogenic commitment and cartilage differentiation, formation of woven bone and osteoclast-mediated remodeling of the callus [11], [12] [11], [12]. In T1DM, there is evidence for decreased progenitor cell proliferation [13]–[17], decreased callus cartilage content [13], [14], [17], [18], premature cartilage resorption [13], [18], [19], decreased callus bone content [14]–[16], [20], [21], and biomechanically inferior repair [14]–[17], [20]. Several groups have demonstrated that administration of sufficient systemic insulin to achieve tight glucose control rescued progenitor cell proliferation, callus bone content, and biomechanical strength [15], [16], [20]. Alternatively, intramedullary insulin delivery rescued the decreased cellular proliferation, cartilage percentage, bone percentage, and mechanical strength that characterize the fracture callus of diabetic rats [14].

Even though T2DM is a much more prevalent and growing disease condition compared to T1DM, the tissue and molecular events that impair fracture healing in T2DM have not been investigated. Here, for the first time, we examine tibia fracture healing in a high fat diet-fed mouse, a classic model of T2DM. Our results demonstrate that the metabolic dysregulation in this model is associated with defective fracture repair that is distinct from that seen in T1DM and is likely related to an aberrant increase in adipocytes in the fracture callus of mice with T2DM.

## Materials and Methods

### Animals

All animal experiments described in this report were reviewed and approved by the University Committee on Animal Resources (IACUC) at the University of Rochester Medical Center. Male C57BL/6J mice (Jackson Laboratories, Bar Harbor, ME, USA) were obtained at 5 weeks of age and housed 5 per microisolator cage in a vivarium housing room on a 12-hour light/dark cycle at the University of Rochester Medical Center. Upon arrival, mice were immediately provided *ad libitum* access to either a control lean diet with 10% total kcal from saturated fat or an experimental high fat diet with 60% total kcal from saturated fat (Catalog Nos. D12450B and D12492 respectively, Open Source Diets, Research Diets Inc., New Brunswick, NJ, USA). The high fat diet (HFD) has been shown to cause weight gain, insulin resistance, and hyperglycemia in this strain of mice [22], [23]. Each mouse was weighed before fracture and again at sacrifice. Glucose tolerance testing (GTT) was performed on representative lean (n = 5) and HFD-fed (n = 5) mice preoperatively [24], [25]. Briefly, mice were fasted for 6 hours, anesthetized with isoflurane (5%) and tail vein blood was sampled using a commercially available glucometer (One Touch Ultra; Lifescan, Inc., Milpitas, CA, USA). A glucose bolus (300 mg/kg) was then injected intraperitoneally. Additional glucose levels were obtained at 15, 30, 60, 90, and 120 minutes, with isoflurane again employed to ensure anesthetic plane for each blood draw. To quantify metabolic status, the net area under the curve (AUC) was calculated from the GTT curve of each mouse (GraphPad PRISM, version 4, GraphPad Software, La Jolla, CA, USA).

### Tibia Fracture Model

Using a model previously established in our Center, we administered open tibia fractures to mice fed either a lean or HF diet [26]. The rationale for using this approach instead of the commonly employed Einhorn method (utilizing the Einhorn device to induce closed fractures via delivery of a blunt force that causes traumatic 3 point bending to initiate failure) is as follows: Our own work [27] as well as studies published by others [28], [29] definitively establish that the HFD induces an osteoclast-dependent bone loss that leads to a weaker skeleton. If we had performed the trauma-induced fracture model developed by Einhorn, there would be a concern that mode of fracture and comminution might be distinct in the diabetic animals due to their skeletal fragility. To avoid this potential complication, and to make the fractures as reproducible as possible across experimental groups, we opted to perform the open tibial fracture osteotomy model, which is a surgical transection of the tibia (without injury to the fibula) that is carefully applied with a scalpel blade. Initial differences in bone strength or fragility do not influence the nature of the injury and insure that at the start of the healing process, the fracture is the same in both control and HFD-fed mice. Since our interest was to further understand the healing process post-fracture, this approach is the best available to factor out bone strength issues that exist pre-fracture. To administer the tibial fractures, mice were anesthetized via isoflurane inhalation (5% induction, 2.5% maintenance). Under sterile conditions, a 15-mm skin incision was made over the anterior aspect of the right lower leg. A 26G, five-eighths inch intradermal needle (BD Medical Systems, Franklin Lakes, NJ, USA) was inserted through the anteromedial tibial plateau to access the medullary canal. This was removed and a 26G Quincke-type spinal needle (BD Medical Systems, Franklin Lakes, NJ, USA) was inserted for trial fit and removed. The mid-diaphysis of the right tibia was identified and a #11 scalpel blade was gently scribed over the posterolateral cortex and slowly drawn back and forth over the developing furrow to complete the osteotomy while preventing comminution. The distal tibia was mobilized to confirm a complete osteotomy. The 26G spinal needle was reinserted to provide intramedullary fixation. The skin was closed using simple 5-0 nylon (Ethicon, Inc., Somerville, NJ, USA) interrupted sutures. Radiographs (Faxitron X-Ray Cabinet, Wheeling, IL, USA) were obtained postoperatively to evaluate the fracture and fixation. Banamine (2.5 mg/kg) or buprenorphine (0.5 mg/kg) was administered for analgesia.

### Micro-Computed Tomography and Microvascular Analysis

Fractured tibiae were harvested at 7, 10, 14, 21, 28, and 35 days postoperatively by disarticulating the right lower-extremity at the knee and cutting the tibia just distal to origin of the fibula. High-resolution micro-computed tomography (vivaCT 40; Scanco Medical AG, Basserdorf, Switzerland) was used to render a three-dimensional image of the tibia. Bone and vascular volumes within the fracture callus were determined by routine methods that we employ regularly in our Center [26], [30], [31] and involves the expertise of our Molecular and Anatomic Imaging Core. Regarding assessment of bone values, samples were scanned using the following parameters: 55 kV energy setting, 300 millisecond integration time, 10.5 µM voxel size, 10 µM slice increment, and a threshold of 210. To analyze external callus bone volume, two contours were created for each slice; the first traced the perimeter of the external fracture callus and the second surrounded all cortical bone from the adjacent uninjured cortices. Subtraction included all mineralized tissues above the threshold between the two contour lines, which encompassed the entire fracture callus. Analysis of vascular volume within the external fracture callus was also performed for selected samples harvested at 10 and 14 days postoperatively. As previously described, lead chromate paint was perfused to visualize all vascular structures [32]. Briefly, mice were anesthetized and a thoracotomy was performed to permit catheterization of the left ventricle and an incision to the right atrium. Approximately 20 mL mixture of 0.9% NaCl and heparin (100 IU/mL) was slowly injected into the left ventricle followed by 20 mL of 10% neutral buffered formalin (NBF). Finally, 4 mL of lead chromate paint (Microfil MV-122 yellow; Flow Tech, Inc., Carver, MA, USA) was injected into the left ventricle and continued until the liver and footpads became yellow. After 30 min at room temperature (RT) carcasses were placed in 10% NBF for 24 hours. Right tibiae were harvested and placed into NBF for 3 days. Tissues were μCT scanned as described above to capture fracture callus bone volume as well as total vascular volume in the callus. Tissues were decalcified in 10% ethylenediaminetetraacetic acid (EDTA) for 3 weeks and then rescanned, which provided the total vascular volume.

### Histology and Histomorphometry

Fractured tibiae harvested for μCT analysis were processed for histology as previously described [33]. Briefly, tibiae were disarticulated at the knee, denuded of soft tissue, cut distally and fixed at RT in 10% NBF for 72 hours. After three washes in phosphate buffered saline (PBS), fixed tibiae were decalcified in 10% EDTA for 7 to 14 days. Tissues were then processed using a Tissue-Tek VIP 6 tissue processor (Sakura Finetek USA, Inc., Torrance, CA, USA) and embedded in paraffin. Serial 3 µm thick sagittal sections were obtained from a 60 µm region spanning the center of the fracture callus. Three sections, each separated by approximately 10 to 15 µm, were stained with Alcian Blue Hematoxylin/Orange G (ABH/OG) and histomorphometric analysis was performed using a point counting method as described previously [34]. Briefly, blinded sections were analyzed using a standardized eyepiece grid under the 10x objective to determine the percent of total callus area composed of cartilage, woven bone, adipocytes, osteoblasts and stromal cells. Each crossing point on the grid was scored to either be cartilage, woven bone, adipocyte or stromal area. Cartilage was defined as tissue with positive Alcian Blue stain. Woven bone was counted whenever a trabecular structure was observed, regardless of staining. Adipocytes were defined as any tissue completely lacking stain and exhibiting a circular or elliptical shape characteristic of adipocytes. Osteoblasts, including stromal cells, were defined as the population that was directly residing bone surfaces that was not multinucleated. Stromal cells were considered to be all cellular tissues within the fracture callus that did not meet the above criteria for cartilage, woven bone or adipocytes or osteoblasts. Cortical bone and internal callus were not included in these analyses. The relative percentage of each tissue type, normalized to the entire callus area as well as the percent of bone perimeter occupied by an osteoblast population was calculated for each section.

### Osteoclast Quantification

One section per sample was stained for tartrate-resistant acid phosphatase (TRAP). Briefly, after deparaffinization and rehydration with distilled water, sections were incubated at 37°C for 25 minutes in a solution of anhydrous sodium acetate (Sigma S-2889), L-(+) tartaric acid (Sigma T-6521), glacial acetic acid, fast red violet LB salt (Sigma F-3381), naphthol AS-MX phosphate (Sigma N-4875), ethylene glycol monoethyl ether (Sigma E-2632), and distilled water. Sections were rinsed in distilled water, counterstained with hematoxylin for 10 sec and then placed in ammonia water for 5 seconds. Quantification was completed using the 10x objective and Osteomeasure software (OsteoMetrics, Inc., Decatur, GA, USA) to contour bone perimeter (B.Pm.) within the anterior callus and identify osteoclasts (Oc.N.), which were defined as multi-nucleated, TRAP-positive cells seated on bone surfaces.

### Immunohistochemical Staining

Immunohistochemistry was performed as described previously [35]. Briefly, an avidin-biotin peroxidase system (Vector Lab, Burlingame, CA, USA) was used to detect two primary antibodies; anti-perilipin rabbit monoclonal antibody (1∶100: Cat. #9349: Cell Signaling Technology, Inc., Danvers, MA) and anti-PPARγ rabbit monoclonal antibody (1∶100: Cat. #2435: Cell Signaling Technology, Inc., Danvers, MA). Reactions were then visualized with diaminobenzidine (DAB) as substrate (Vector Lab, Burlingame, CA, USA). The sections were counterstained with hematoxylin. All staining procedures were followed as instructed by the manufacturer.

### Biomechanical Testing

Biomechanical properties of healed fractures were assessed at 14 and 35 days postoperatively as described previously [36]. Briefly, right tibiae were harvested, immediately flash-frozen in liquid nitrogen and then stored at −80°C. Prior to testing, samples were thawed over 30 minutes at RT and sent for μCT scanning. After μCT, polymethylmethacrylate (PMMA) bone cement (Endurance Cement; Depuy Orthopaedics, Inc., Warsaw, IN) was used to cement tibiae into square 6.35 mm^2^ aluminum sleeves, taking care to align the long axis of each tibia with the sleeve's center of rotation. Samples were rehydrated and loaded into an EnduraTec TestBench TM System (200 Nmm torque cell; Bose Corporation, Minnetonka, MN, USA) and tested in torsion at a rate of 1°/s until failure. Torque data were plotted against rotational deformation and maximum torque at the point of failure was recorded for each sample. All samples were blinded throughout preparation and testing.

### Statistical Analyses

Data presented are the mean and standard error for lean and HFD-fed mice, unless specified otherwise. Two-tailed, unpaired Student's *t* tests were calculated using Excel software (Microsoft Corporation, Redmond, WA, USA). Two-way ANOVA tests were calculated using GraphPad PRISM software, (GraphPad Software, La Jolla, CA, USA). Significance is indicated using asterisks; *p<0.05, **p<0.01, and ***p<0.001.

## Results

### Induction of obesity and glucose intolerance by HFD

**Figure 1:**
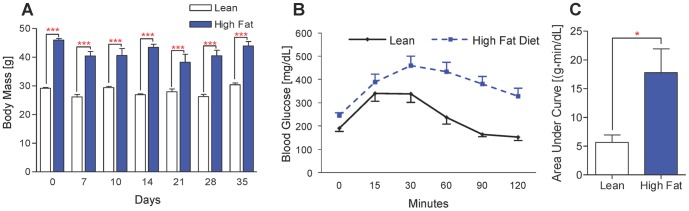
HFD-fed mice are obese and glucose intolerant. (**A**) Body weights of HFD-fed and control lean diet-fed mice at various time points after surgical fracture. Bars represent means ± SEM (n≥5). ***p<0.001 using a two-way, unpaired ANOVA with a Bonferroni post-test. (**B**) Glucose tolerance testing (GTT) was performed immediately prior to fracture on lean and HFD-fed mice. Plotted data points represent means ± SEM (n = 5). (**C**) Net area under the curve (AUC) was calculated for each mouse that underwent GTT (n = 5). *p<0.05 compared to respective lean diet controls using an unpaired, two-tailed Student's *t* test. Bars represent means ± SEM (n = 5).

To confirm that HFD-fed mice recapitulated key pathogenic features of human T2DM, we weighed all mice and performed glucose tolerance testing (GTT) on a representative group. After twelve weeks of *ad libitum* access to the assigned diet, the HFD-fed mice weighed significantly more than lean controls (*p*<0.001). Importantly, HFD-fed mice remained significantly heavier throughout the postoperative period compared to time-matched lean controls (Fig. 1A, *p*<0.001). GTT performed preoperatively showed that HFD-fed mice were unable to restore basal blood glucose levels 120 minutes after glucose bolus while lean-fed control mice restored blood glucose levels after 90 minutes, indicating that HFD-fed mice had impaired glucose handling, which is characteristic of human patients with T2DM (Fig. 1B) [24]. This was confirmed by calculating the net area under the curve (AUC) for each mouse. HFD-fed mice had a significantly larger net AUC preoperatively, indicating impaired glucose handling (Fig. 1C, *p*<0.05). Taken together, these results confirm that our HFD-fed mice recapitulate two important features of T2DM in human patients.

### HFD does not impair neovascularization of the fracture callus

**Figure 2:**
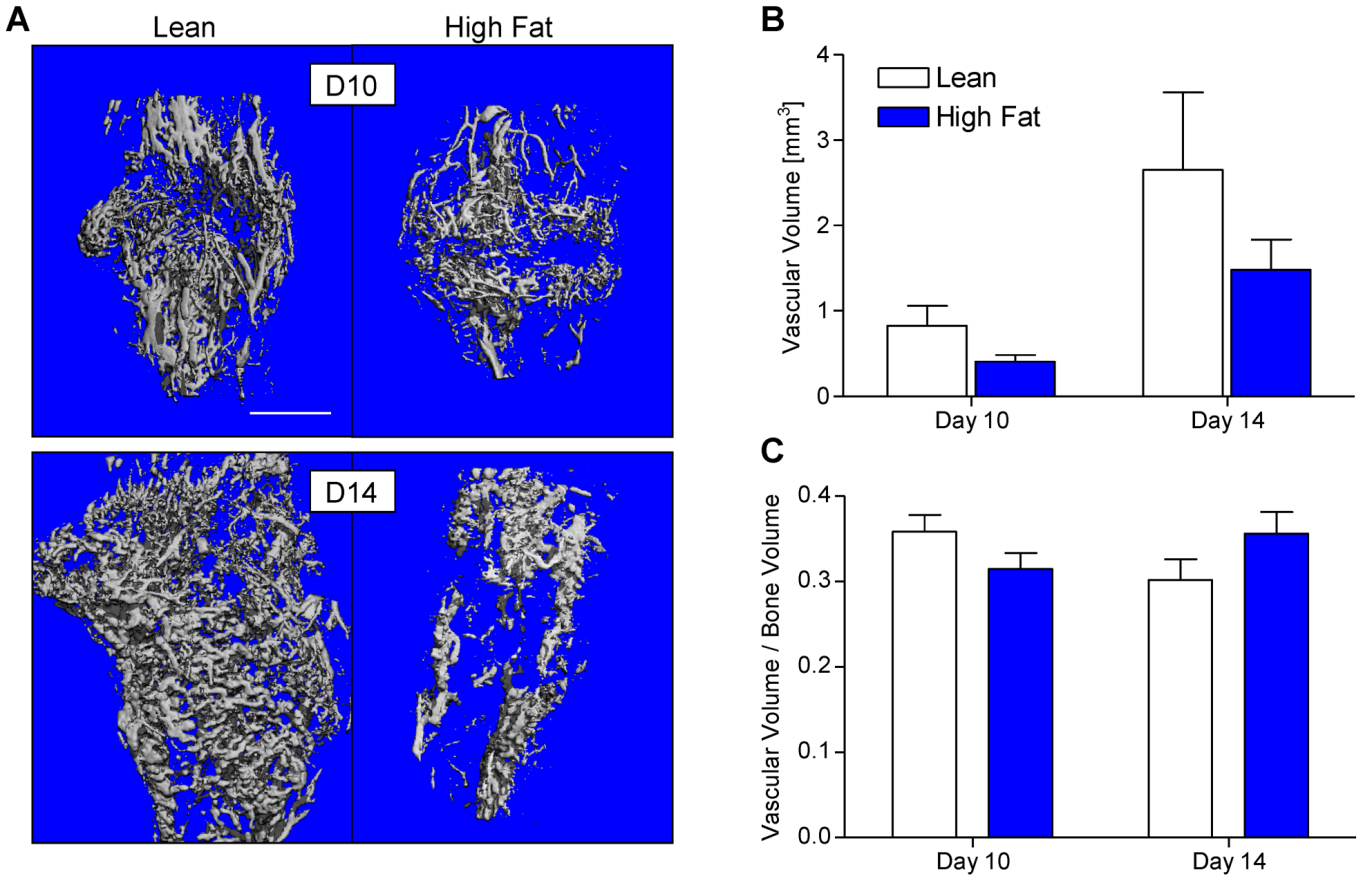
Callus neovascularization in lean- and HFD-fed mice is similar. μCT scans of fractured tibiae that were perfused with lead chromate paint after decalcification were performed in lean and HFD-fed mice at 10 and 14 days post-fracture. Representative three-dimensional reconstructions are presented in panel (**A**). Vascular volume (**B**) and vascular volume normalized to callus bone volume (**C**) was quantified from μCT data. Bars represent means ± SEM (n≥5). Scale bar (white line)  = 1 mm.

Human T2DM is characterized by numerous microvascular complications leading to retinopathy [37], renal dysfunction [38], and peripheral neuropathy [39]. Furthermore, in rodent models of fracture healing in the context of T1DM, reduced callus vascularization during healing has been documented [40], [41]. Thus, we performed lead chromate perfusion-based vascular μCT at days 10 and 14 to investigate whether HFD-fed mice exhibited impaired neovascularization of the fracture callus. Serial μCT scanning was performed to capture bone and lead chromate volume prior to decalcification and a second scan after decalcification yielded lead chromate volume within vascular spaces. Representative three-dimensional reconstructions from μCT scans after decalcification show a qualitative trend towards decreased callus vascular volume in HFD-fed mice (Fig. 2A). Quantification confirmed this trend but significance was not achieved at either time point (Fig. 2B). This trend towards decreased callus vascularity in HFD-fed mice was not apparent after normalizing vascular volume to bone volume for each sample (Fig. 2C), indicating that apparent reduced vascularity in the calluses was likely associated with a net reduction in callus. This experiment confirms the positive correlation between vascular volume and bone volume but a definitive link between reduced fracture callus vascular volume and feeding of a HFD cannot be established from this experiment.

### HFD-fed mice display decreased fracture callus bone content in late-stage healing

**Figure 3:**
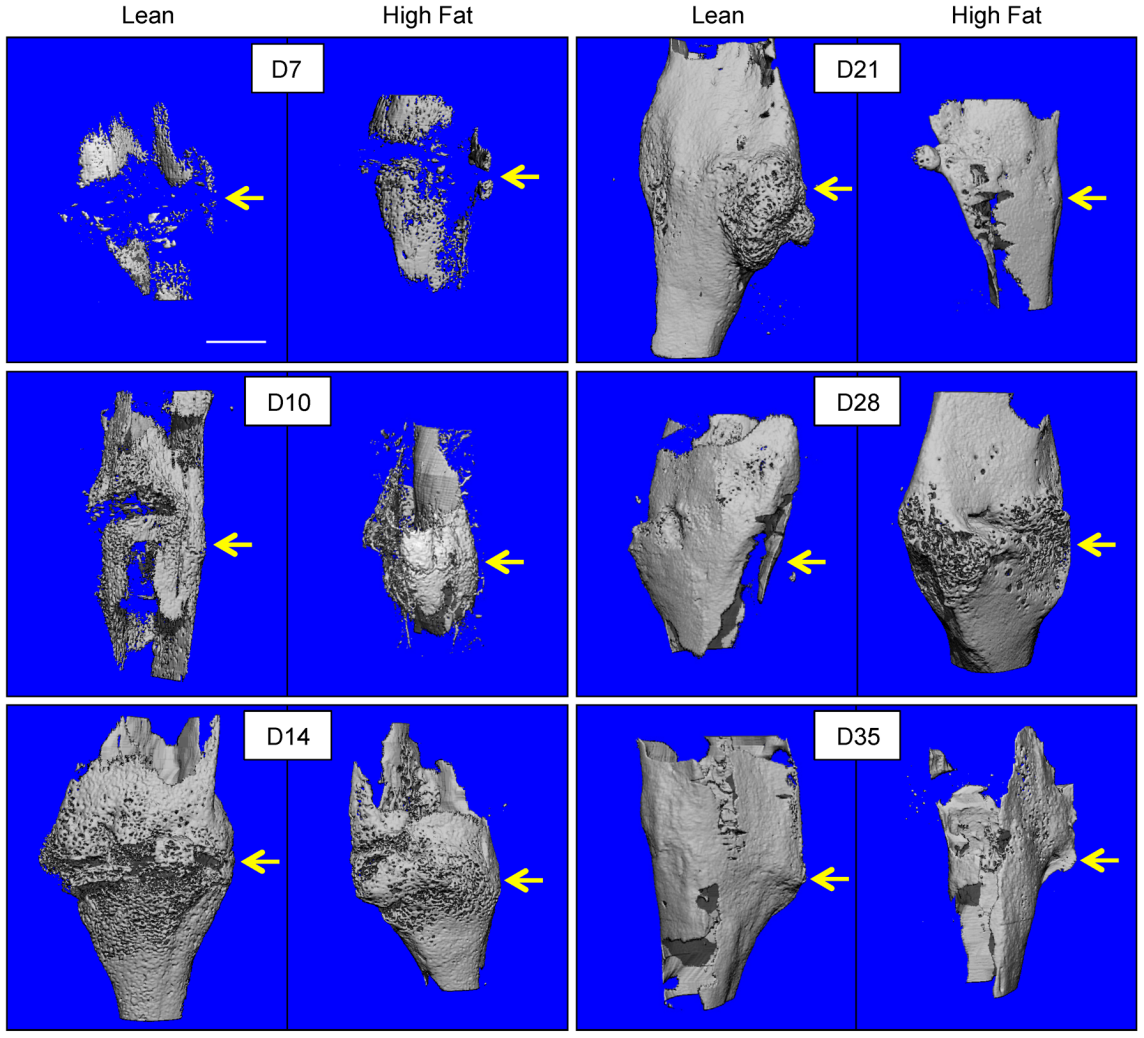
μCT imaging reveals a trend toward decreased bone volume in HFD-fed mice. μCT scans were performed on tibiae at 7, 10, 14, 21, 28, and 35 days post-fracture. Representative three-dimensional reconstructions, from groups of 5–7 tibiae, show bone volume within the external fracture callus. Scale bar (white line)  = 1 mm. Fracture sites are denoted with yellow arrows.

**Figure 4:**
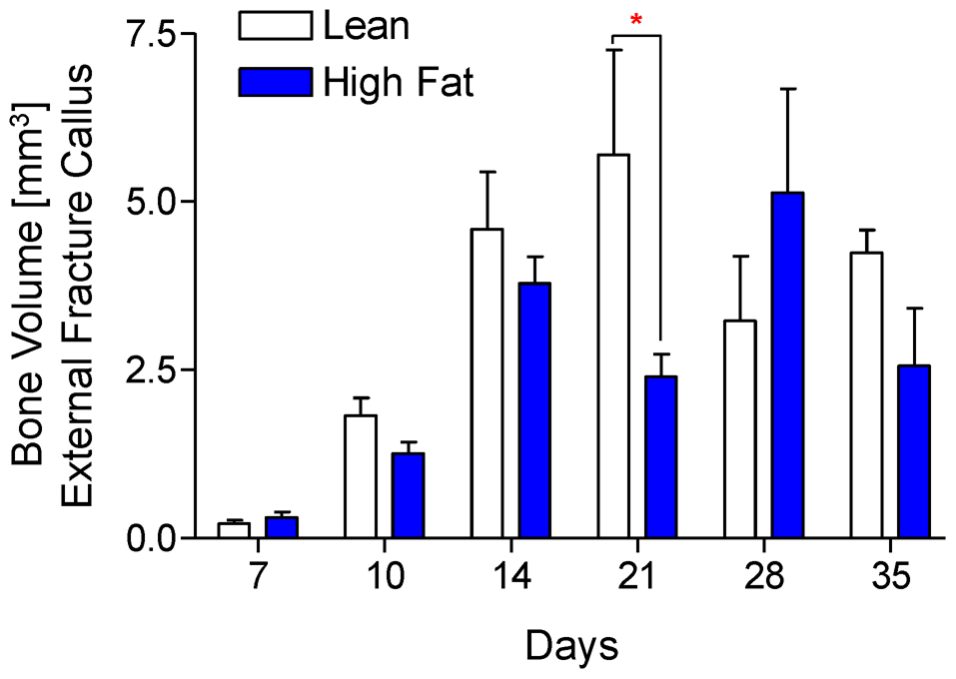
Quantitative analysis confirms decreased and delayed accrual of peak bone volume in fracture callus of HFD-fed mice. Using μCT scans that were used create the reconstructions shown Fig. 3, bone volume was quantified from lean- and HFD-fed mice at 7, 10, 14, 21, 28, and 35 days post-fracture. Bars represent means ± SEM (n≥5). *p<0.05 compared to time-matched lean diet controls using two-way, unpaired ANOVA with Bonferroni post-tests.

**Figure 5:**
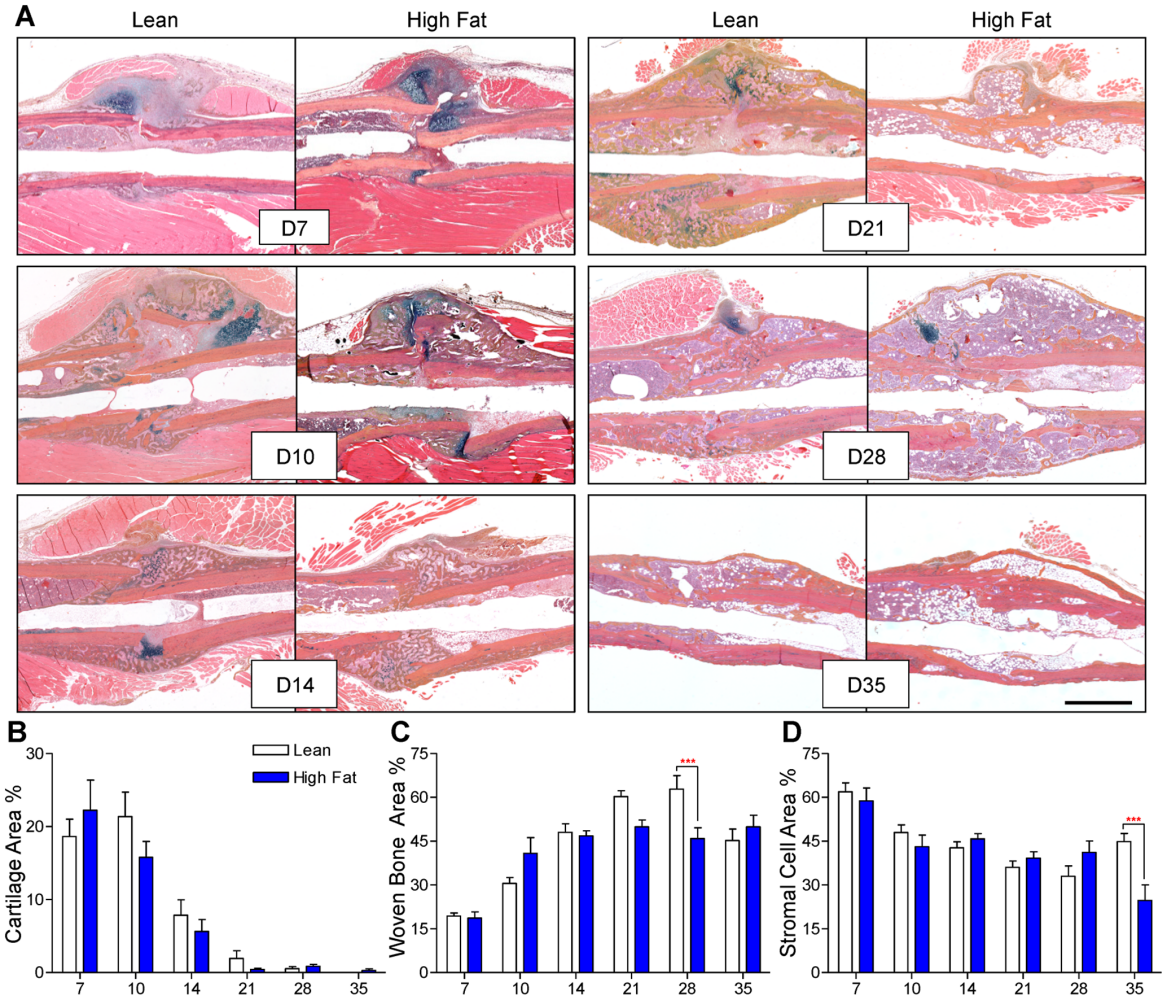
Woven bone content is decreased at later stages of fracture healing in HFD-fed mice. (**A**) Representative Alcian Blue Hematoxylin/Orange G stained histologic sections of fracture callus in lean- and HFD-fed mice at 7, 10, 14, 21, 28, and 35 days post-fracture. Quantification of cartilage (**B**), woven bone (**C**), and stromal cell areas (**D**), expressed as percentages of total callus area, were determined using a point-counting histomorphometric method. Bars represent means ± SEM (n≥5). ***p<0.001 compared to time-matched controls using two-way, unpaired ANOVA with Bonferroni post-tests. The black size marker in the lower right panel  = 1 mm.

Fracture callus bone volume was determined via μCT studies performed at various time points during healing. Representative μCT reconstructions are presented for all time points (Fig. 3), with the correlated quantitation shows significantly decreased bone volume at day 21 and an apparent delay in achieving peak bone volume and in HFD-fed mice compared to lean-fed controls (Fig. 4). Alcian Blue Hematoxylin/Orange G-stained histologic sections (Fig. 5A) were used to perform histomorphometry-based study of the callus, revealing no differences in cartilage content between the groups at any timepoint (Fig. 5B), but confirming decreased bone content, with HFD-fed mice showing a significant decrease in woven bone area as a percentage of total callus area at day 28 (Fig. 5C). Interestingly, HFD-fed mice showed significantly decreased stromal area at day 35, which may be due to expansion of the adipocyte population with possible suppression of bone marrow stem cells and/or other progenitor or stromal cell populations (Fig. 5D). It should be noted that TRAP-staining to examine ratio of osteoclast number to bone perimeter was performed, indicating that the ratio of osteoclast number (Oc.N.) to bone perimeter (B.Pm.) was not different between lean- and HFD-fed mice at any time point (data not shown).

### HFD-fed mice display increased fracture callus adiposity and decreased osteoblast-occupied bone surface area

**Figure 6:**
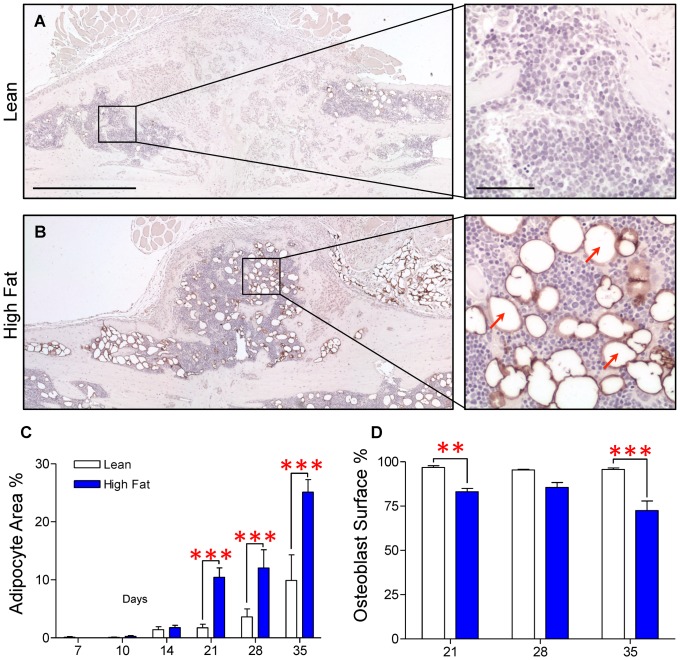
Adiposity is increased and osteoblast-occupied bone surface is decreased within the external fracture callus during late stage healing in HFD-fed mice. Immunohistochemical staining for perilipin was performed to confirm the presence of adipocytes within the fracture callus. Representative sections from day 21 post-fracture demonstrate an increased number of adipocytes within the fracture callus of the HFD-fed mouse (**B**) compared to the lean diet control (**A**). Higher magnifications of selected regions from lean and HFD-fed calluses illustrate specificity of perilipin staining, with several typical perilipin-positive adipocytes denoted with red arrows. (**C**) Adipocyte area as a percentage of total callus area and percent of osteoblast occupied bone surface were determined using histomorphometric methods in lean- and HFD-fed mice at the indicated time points. Bars represent the means ± SEM (n≥5). **p<0.01 and ***p<0.001 compared to time-matched controls using an unpaired, two-way ANOVA with Bonferroni post-test. The black size marker in the low magnification images (left panels of **A** and **B**)  = 1 mm. The black size marker for the high magnification images (right panels of **A** and **B**)  = 100 µm.

Within the fracture callus, cells with spherical morphology and lacking intracellular organelles were observed. These presumptive adipocytes were markedly more abundant in fracture calluses of HFD-fed mice (Fig. 6B) than in those of lean diet controls (Fig. 6A). To confirm that these cells were adipocytes, immunohistochemical staining was performed using a monoclonal antibody for perilipin, a protein associated with the membranes of lipid vacuoles within adipocytes [42]. As shown in Fig. 6B, the ribbon-like lipid vacuole membranes within presumptive adipocytes specifically stained for perilipin, confirming that these are adipocytes. Conversely, the fracture calluses of lean diet control mice exhibited a paucity of perilipin-positive adipocytes (Fig. 6A). Correlated with this, histomorphometry revealed that adipose tissue area as a percentage of total callus area was significantly increased in HFD-fed mice at days 21, 28 and 35 (Fig. 6C). Interestingly, adipose tissue was minimal at early time points through day 14 in both HFD-fed and lean control mice. Increased adiposity was observed in the HFD-fed mice only at day 21 and the later time points. This change in adipocyte area was coincident with a significant decrease in percentage of bone surface occupied by osteoblast-like cells in HFD-fed mice, an effect that was significant at day 21 and 35 post-fracture, with a trend toward a decrease at day 28 (Fig. 6D). Taken together, these data establish that during the later stages of healing, HFD-fed mice have a concomitant increase in callus adiposity, and a decrease in woven bone content/osteoblast-occupied bone surface suggesting a single pathophysiological process may be responsible for both defects.

### PPARγ expression is up-regulated in the callus of HFD-fed mice

**Figure 7:**
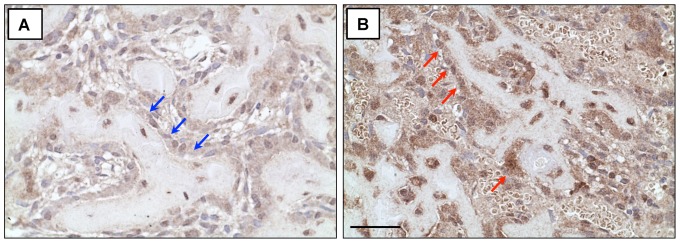
PPARγ expression is increased in fracture callus of HFD-fed mice. Sections were subjected to immunohistochemical staining for PPARγ and representative fracture calluses at day 14 show increased PPARγ expression in the fracture callus of HFD-fed mice (**B**) compared to lean controls (**A**), particularly in cells adjacent to trabecular bone elements (marked by blue arrows in callus from lean-fed mice and red arrows in callus from HFD-fed mice). The black size marker (panel **B**)  = 20 µm.

Given the increase in the number of adipocytes in fracture callus from HFD-fed mice, we further examined the expression of peroxisome-proliferation activated receptor, subtype gamma (PPARγ), widely recognized as a master regulator of adipogenesis [43], [44]. Since we observed callus adipocyte number to increase at time points from days 21 through 35 post-fracture (Fig. 6C), we chose to perform PPARγ immunohistochemistry at the previous time point (day 14), where PPARγ might be expected to be up-regulated to drive the commitment of progenitor populations toward adipocyte formation. While PPARγ expression was detectable in day 14 fracture callus from both lean- (Fig. 7A) and HFD-fed (Fig. 7B) mice, the HFD-fed group had increased PPARγ staining overall, particularly in cells adjacent to trabecular bone (Fig. 7B, red arrows), compared to lean diet controls (Fig. 7A, blue arrows).

### Biomechanical strength during late-stage healing was decreased in HFD-fed mice

**Figure 8:**
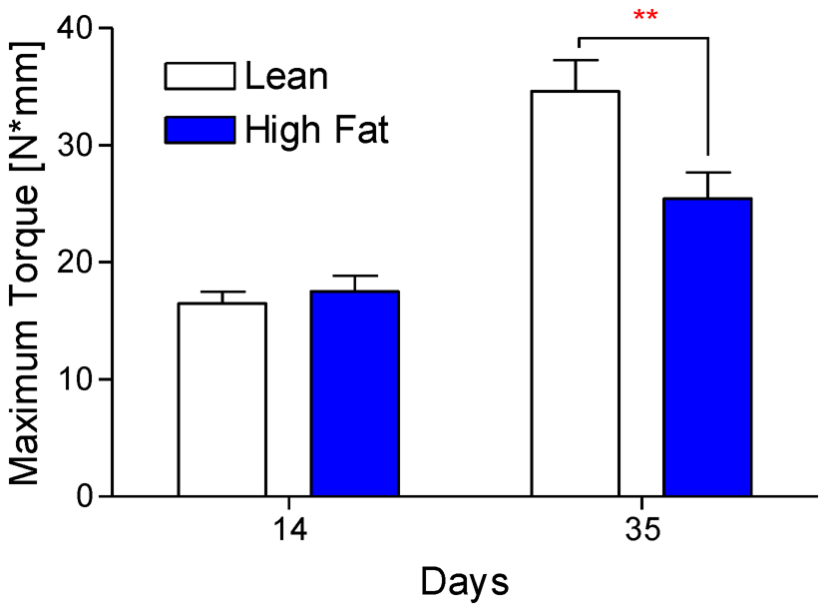
Healed tibiae from HFD-fed mice are biomechanically weaker. (**A**) Surgically fractured right tibiae from lean- and HFD-fed mice (n≥8) were subjected to biomechanical torsional testing at days 14 and 35 post-fracture, with a significant decrease in strength observed in the HFD-fed group at day 35. Bars represent the means ± SEM (n≥8). **p<0.01 compared to time-matched controls using an unpaired, two-way ANOVA with Bonferroni post-tests.

Torsional testing was performed on surgically fractured tibiae at days 14 and 35. HFD-fed mice had significantly weaker healed fractures at day 35 (Fig. 8), but no difference in maximum torque to failure was observed at day 14. This finding corresponds with our data that shows no differences in woven bone or adipocyte percentage between HFD-fed and lean control diet mice until day 21.

## Discussion

Endochondral-based healing, which predominates in repair of long bones, requires the correct temporospatial coordination of a series of molecular and cellular events [45]. From a tissue architecture perspective, these events are organized into four overlapping phases: (1) inflammation, (2) soft (cartilaginous) callus formation, (3) hard (woven bone) callus formation, and (4) woven bone remodeling. Mesenchymal cells (MSCs) and chondro-/osteo-progenitor populations that primarily reside in the periosteum are essential throughout fracture repair [45]–[49]. Angiogenesis and neovascularization are required as a conduit for MSC delivery during hard callus formation and to provide oxygen tension sufficient to induce MSCs to differentiate into osteoblasts [4], [11], [12], [45], [47]. From the perspective of the work presented in this report, it is interesting to note that osteoblastic progenitors [11] and preadipocytes [44] have both been identified as populations of progenitor cells whose source is perivascular. Overall, it is generally held that fracture healing is impaired when MSCs are dysfunctional, which can include a decreased progenitor pool, ineffective recruitment, aberrant fate determination, or impaired differentiation.

Patients with T1DM and T2DM sustain pathologic changes to normal skeletal homeostasis and repair. Osteopenia and osteoporosis, human disease states characterized by decreased bone mineral density and/or bone quality, are more prevalent in patients with either type of DM [50]. Furthermore, the constellation of pathologic changes that accumulate in patients with DM coalesce to impair skeletal repair, manifesting as an increased incidence of delayed healing or fracture non-union [8] and increased rates of pseudoarthrosis after surgical arthrodesis [51]–[53]. Work focused on understanding the impact of T1DM on skeletal repair has been published previously in rodent models of tibia and femur fracture following STZ-dependent pancreatic beta cell depletion. As mentioned, these studies have revealed that key defects include decreased progenitor cell proliferation [13]–[17], decreased callus cartilage content [13], [14], [17], [18], premature cartilage resorption [13], [18], [19], reduced neovascularization of the callus [40], [41], decreased callus bone content [14]–[16], [20], [21], and a biomechanically inferior repair at later time points [14]–[17], [20]. Based on these clinical and animal data establishing an impairment of bone healing in DM, and because T2DM in particular is highly prevalent and a major health crises in the US and worldwide, we characterized the fracture healing process in mice induced to have obesity-related type 2 diabetes that was induced via consumption of a high fat Western diet. We hypothesized that the deleterious effect of T2DM on the bone healing process involves an alteration in one or more of the phases of healing.

Findings presented here establish that obesity-associated T2DM leads to defective fracture repair that is distinct from the defect seen in T1DM from the perspective of early callus tissue morphogenesis. The two most striking differences relate to neovascularization of the callus during early healing and induction of osteoclast formation and numbers during mid-late healing. Regarding the vascular phenotype, while a key defect in T1DM is reduced cartilage content [13], [14], [17], [18] and a correlated impairment in callus neovascularization [40], [41], T2DM did not significantly impair the cartilage phase of healing or the subsequent propagation of vascular tissue within the callus. Since it is established that chondrocyte-dependent production of VEGF is a critical signal for vascular ingrowth during bone repair [54], the reduced cartilage phenotype in T1DM is consistent with the decreased neovascularization observed in that model. Conversely, a normal cartilage phase in T2DM, which would presumable include appropriate expression of VEGF, may account for the absence of a vascular phenotype in this model. This tissue dynamic (i.e. chondrocyte hypertrophy leading to VEGF-dependent neovascularization) is likely unique to bone healing, and may explain why impaired vascularization in other situations, such as during dermal wound healing [55], [56], is a phenotype shared by T1DM and T2DM. Regarding the osteoclast phenotype, it is established that in T1DM, increased levels of TNF-α lead to increased osteoclastogenesis and a higher number of osteoclasts [19], [57], which may account for the accelerated resorption of the cartilage callus during mid-stage healing in this model. This phenotype is not observed in T2DM based on our assessment of osteoclast number, an analysis that revealed no differences between mice fed the normal versus the HFD. It may be that the complete loss of insulin in T1DM leads to molecular and tissue effects during healing that are more robust and/or distinct from the impact of the diet-induced insulin resistance (i.e. incomplete ablation of insulin sensitivity or insulin production) that occurs in T2DM. It has been shown that osteoclast numbers/function during skeletal homeostasis is enhanced in T2DM [28], and that this effect could lead to obesity-related bone loss [29], but results presented here suggest that in the stressed situation present during fracture repair, the osteoclast phenotype is unaffected. Understanding the molecular and tissue basis for this distinction is an important focus for ongoing study.

Further differences in bone repair between T1DM and T2DM are also seen in the post-cartilage phases of healing within the callus. While T1DM shows late stage healing defects that likely result from the initial soft callus defects (reduced cartilage/neovascularization), the central tissue defect we observed in T2DM is a significant increase in fracture callus adiposity in HFD-fed mice during the woven bone formation and remodeling phases that was coincident with a decrease in both the area of osteoblast-occupied bone surface and callus bone volume during mid to late healing. This ultimately led to a biomechanically weaker repair at day 35 post-fracture. Interestingly, adipocytes were not appreciably observed within the fracture callus until day 14, with calluses from both lean and HFD-fed mice up through this time point possessing a minimal and approximately equal number of adipocytes. After 14 days, a significant increase in adipose tissue content was observed in calluses from HFD-fed mice, coinciding with the period of maximal woven bone formation and subsequent remodeling driven by osteoblast formation and osteoblast-osteoclast coupling respectively. The simultaneous increase in adipose tissue and decrease in osteoblast-occupied surface area and woven bone content in HFD-fed mice compared to lean diet controls suggests a potential MSC fate switch, with a possible shift toward the adipocyte lineage at the expense of osteoblastic commitment. This idea that fate switching drives the phenotype is further supported by the observation of increased PPARγ expression within the fracture callus of HFD-fed mice, particularly in the population of cells lining bone trabeculae.

Mounting evidence for the mutual exclusivity of bone and adipose tissue formation suggests a reciprocal regulation of MSC fate with respect to adipogenesis and osteoblastogenesis [46], [58]–[60]. PPARγ, one of 3 known subtypes of peroxisome-proliferation activated receptors (PPARs), which are transcription factors of the steroid/thyroid hormone receptor superfamily [61], [62], has traditionally been regarded as the master regulator of adipogenesis and has been implicated as a key regulator that drives MSCs down either the adipocytic or osteoblastic lineage [43], [63]. This idea has been most definitively demonstrated in animal studies of PPARγ signaling and its influence on skeletal biology. Akune et al. deleted the PPARγ gene in mice to characterize its role in the regulation of adipogenesis and osteoblastogenesis. Embryonic stem (ES) cells cultured from PPARγ^−/−^ embryos in medium lacking osteogenic stimulation demonstrated a complete absence of adipogenesis coupled with increased bone nodule formation [59]. Col1α1, osteocalcin, and Runx2 expression were significantly increased in PPARγ^−/−^ ES cells compared to WT ES cells, providing molecular evidence of fate switching in the cultures. These findings correlate with in vivo data from PPARγ^+/−^ mice demonstrating increased bone volume and expression of osteoblast markers in conjunction with decreased adipocyte volume and expression of adipocyte markers [59]. Furthermore, epidemiological data suggest that PPARγ agonists from the thiazolidinedione (TZD) family of drugs adversely affect skeletal homeostasis with multiple large, prospective clinical trials demonstrating an excess fracture risk among diabetic patients treated with TZDs [64]–[66]. The TZDs have been used extensively to treat patients with T2DM [64], [67] and the excess fracture risk in patients exposed to TZDs is likely due to the development of osteoporosis. While these observations do not speak directly to fracture healing, they do support the idea that PPARγ likely plays an important role in the balance between the osteoblastic and adipogenic pathways [46], [58]–[60]. Overall, these results suggest that PPARγ, either independently or in concert with other emerging candidate signaling regulators of adipogenesis, such as the non-canonical Wnt signaling pathway [68], plays a central role in balancing adipogenesis and osteoblastogenesis. This concept is consistent with the increased PPARγ expression and the increased adiposity/decreased osteoblast number and woven bone content in mid- to late-stage healing that we observed in T2DM.

Data presented in this report support the concept that adipogenesis and osteoblastogenesis are interrelated in the context of fracture repair. Normally, a balance between these fates exists during healing, with the osteoblast fate the dominant outcome. Osteoblastic commitment supports woven bone formation and the downstream production of osteoblast-produced factors known to regulate osteoclast formation (RANKL and osteoprotegerin) and callus remodeling. Based on our findings, we propose that the balance of progenitor cell differentiation is partially shifted toward the adipocyte lineage in HFD-fed mice. This results in an increase in adipocyte formation and an associated decrease in osteoblast commitment. Based on immunohistochemistry findings, we propose that increased PPARγ expression may contribute to this altered fate for progenitor cells leading to the healing defect seen in the HFD-fed population.

As mentioned, insulin has known anabolic effects on bone, with both systemic and local delivery improving fracture repair in T1DM animal models [13]–[15], [19]. Furthermore, clinical reports suggest that better glycemic control, as measured by lower hemoglobin A1c percentage, results in fewer complications of diabetes mellitus [69]. Of specific relevance to the work presented here, rates of healing after transmetatarsal foot amputation were significantly lower in diabetic patients with poorly controlled disease (HbA1c ≥8%), compared to diabetic patients with better control (HbA1c <8%) [70]. Based on these observations and our current work, we suggest that a method to improving fracture repair in patients with T2DM, with the potential to normalize the balance between adipogenesis and osteoblastogenesis in the callus, would be to employ insulin therapy to control the diabetes during the healing process. While multiple and distinct mechanisms have been implicated in mediating diabetic complications in various target tissues, intensive insulin therapy has been shown to be effective in decreasing the frequency and severity of most diabetic complications. Other than insulin, it will require further investigation to identify novel therapeutic approaches, including strategies to inhibit adipogenesis in the fracture callus, to address impaired fracture repair in type 2 diabetes.
